# Psychometrics assessment of ethical decision-making around end-of-life care scale for adolescents in the final stage of life

**DOI:** 10.3389/fped.2023.1266929

**Published:** 2024-01-22

**Authors:** Fateme Mohammadi, Seyedeh Zahra Masoumi, Salman Khazaei, Seyyed Mohammad Mahdi Hosseiny

**Affiliations:** ^1^ School of Nursing and Midwifery, Chronic Diseases(Home Care) Research Center and Autism Spectrum Disorders Research Center, Department of Nursing, Hamadan University of Medical Sciences, Hamadan, Iran; ^2^ Department of Midwifery, School of Nursing and Midwifery, Mother and Child Care Research Center, Hamadan University of Medical Sciences, Hamadan, Iran; ^3^ Health Sciences Research Center, Health Sciences & Technology Research Institute, Hamadan University of Medical Science, Hamadan, Iran; ^4^ Department of Pediatrics, School of Medicine, Besat Hospital, Hamadan University of Medical Sciences, Hamadan, Iran

**Keywords:** psychometrics, ethics, decision-making, end stage, adolescent

## Abstract

**Introduction:**

Healthcare professionals have a critical role in ethical decision-making around end-of-life care. Properly evaluating the ethical decision-making of health care professionals in end-of-life care requires reliable, tailored, and comprehensive assessments. The current study aimed to translate and assess psychometrically a Persian version of the ethical decision making in end-of-life care scale for Iranian adolescents in the final stages of life.

**Methods:**

The present study investigates the methodology and multicenter research. 310 healthcare professionals who treat/care for adolescents at the end of life were selected from 7 cities in Iran. The original version of the end-of-life care decision-making scale was translated into Persian using the forward-backward translation method, and its psychometric properties were evaluated using COSMIN criteria.

**Results:**

Exploratory factor analysis revealed that the factor loadings of the items ranged from 0.68 to 0.89, all of which were statistically significant. Furthermore, three factors had eigenvalues greater than 1, accounting for 81.64% of the total variance. Confirmatory factor analysis indicated a proper goodness of fit in the hypothesized factor structure. The internal consistency reliability of the tool was assessed in terms of its homogeneity, yielding a Cronbach's alpha coefficient of 0.93.

**Conclusion:**

The Persian version of the End-of-Life Care Decision-Making Scale demonstrates satisfactory validity and reliability among healthcare professionals working with adolescents in the final stages of life. Therefore, nursing managers can utilize this tool to measure and evaluate ethical decision-making in end-of-life care for adolescents in the final stages of life and identify the most appropriate strategies, including educational interventions, to improve ethical decision-making in end-of-life care if necessary.

## Introduction

Professional ethics is an inherent aspect of the medical profession, as the mission of various healthcare professions is to provide healthcare, nursing, treatment, and rehabilitation services at the highest standard to ensure, maintain, and promote the health of individuals in society (1). Therefore, healthcare and nursing professions are based on ethics (2). Accordingly, the American College of Physicians lists friendship, excellence, dignity, honesty, respect for patients, and responsibility as the fundamental components of professional ethics in medicine (3). Among these, one of the most crucial topics in professional ethics is responsibility and ethical decision-making in patient care (4). Ethical decision-making is an organized form of ethical reflection to resolve ethical conflicts (5). Meanwhile, healthcare professionals encounter numerous ethical conflicts in challenging clinical environments, influencing their ethical decision-making (4, 5).On the other hand, while considering logic and emotion in ethical decision-making, healthcare professionals must also respect patients’ ethical rights (6, 7). In this context, the ethical decision-making of healthcare professionals is significantly influenced by individual and organizational characteristics such as staff shortages, organizational limitations, knowledge, experience, intellect, cognitive abilities, and ethical sensitivity (8, 9).

On the other hands, the failure to observe ethics and ethical incorrect decisions can lead to medical malpractice, loss of clinical privileges or medical, injury to the patient, discomfort, dissatisfaction and distrust to the staff (10). Therefore, it is necessary for healthcare professionals to be aware of blind spots that may affect their ethical decisions (11, 12). Ethical blind spots is defined as a person's temporary inability to see the ethical aspect of a decision they are making (12). It is often caused by external factors due to which an individual is unable to see the immoral aspect of their behavior in that particular situation (12, 13). One good way to avoid blind spots is to focus on patients as individuals (11). So, Health care professionals should speak with patients and be aware about their cultural backgrounds, religious values and beliefs, thoughts, ethnic values, social norms and their stress and tensions (11, 12). Because if professionals are not aware of blind spots, they may recommend treatments that are against a patient's wishes and lead to wrong and unethical decisions severely affect professional performance (11, 14).

End-of-life care is one of healthcare professionals' most challenging ethical decision-making situations (15, 16). This type of care involves a broad spectrum of physical, psychological, and supportive services for patients and their families (16). The ethical care and decision-making of healthcare professionals for patients in end-of-life care can be significantly influenced by their beliefs, attitudes, work experience, and social factors (17, 18).

Caring for adolescents in the end-of-life stages is particularly challenging (19) because death is a frightening and terrifying experience for them, causing them to express various fears about life after death, parental abandonment, and being left alone (20). As a result, they may question caregivers and parents or engage in fantasies to control their fears and anxieties about death (13). On the other hand, teenagers are a particular sensitive group as patients may become adults and may have different goals of care than their parents (21). Therefore, healthcare professionals must take immediate ethical interventions and decisions to promote family cohesion, reduce death-related anxiety, and help adolescents and their families cope with death (18, 20).

Therefore, assessing and evaluating the ethical decision-making ability of healthcare professionals, especially those caring for adolescents in the end-of-life stages, is essential. In this regard, the Nurses' Ethical Decision-Making around End of Life Care Scale (NEDM-EOLCS) is one of the most practical scales for assessing the ethical decision-making ability of nurses and healthcare professionals. This scale was developed by Kim et al. in 2011 in Korea and included 55 items across three dimensions of thical responsiveness, ethical reasoning, and ethical performance. The tool has appropriate validity and reliability in Korea and has been used to assess the ethical decision-making ability of healthcare professionals (22, 23).

However, there is no standardized tool in Iran to evaluate precisely the ethical decision-making ability of healthcare professionals caring for adolescents in the end-of-life stages. As a result, the ethical decision-making ability of healthcare professionals is not accurately measured, hindering the identification of their strengths and weaknesses in ethical decision-making and potentially leading to unethical decisions. Given the significant importance of assessing and evaluating the ethical decision-making ability of healthcare professionals caring for adolescents in the end-of-life stages and the lack of a valid and reliable tool in Iran.

However, beliefs, values, culture, religion and social norms are very affect in the design and development of tools, especially psycho-cognitive scales. Therefore, when a tool is to be used in a population and a society with a different culture, it is necessary that to psychometric according to the society and culture (24). Therefore, this study aimed to translate and psychometrically evaluate the Persian version of the Nurses' Ethical Decision-Making around End of Life Care Scale for healthcare professional in Iran. Also, the research question in this study was “how is the validity and reliability of the Nurses’ Ethical Decision-Making around End of Life Care Scale for use in Iranian society”.

## Methods

### Study design and setting

This methodological study was conducted in 2023 in 11 hospitals of seven cities in Iran to evaluate the psychometric properties of the Nurses' Ethical Decision-Making around End of Life Care Scale (NEDMEOLCS). The study aimed to assess the face and content validity of the scale, perform exploratory and confirmatory factor analysis, and evaluate the scale's reliability regarding internal consistency and stability. The ethical decision-making around the end-of-life care scale (NEDM-EOLCS) underwent an evaluation of its psychometric properties utilizing the COSMIN (Consensus-based Standards for the selection of health Measurement Instruments) criteria.

### Participants

The sample size required for evaluating the psychometric properties of NEDM-EOLCS was initially determined based on the number of inventory sections, resulting in a recommendation of 5–10 subjects per item (24, 25). However, in this study, a larger sample size of approximately 5 respondents per item was chosen through convenience sampling of in 11 hospitals of seven cities to ensure greater accuracy in both exploratory and confirmatory factors. To be more specific, participants were selected through convenience sampling from seven hospitals. A total of 310 healthcare professionals participated in the exploratory factor analysis, and a separate group of 310 healthcare professionals participated in the confirmatory factor analysis, with no overlap between the two staps. The inclusion criteria for both groups were to have a bachelor's degree, master's degree or PhD degree in nursing, working in a hospital and working often with end-of-life patients and having at least one year of work experience.

### The ethical decision-making around the end-of-life care scale (NEDM-EOLCS)

The self-reported questionnaire of Ethical Decision-Making around End of Life Care Scale (NEDM-EOLCS) is in English (1, 5). The 55 items of the NEDM-EOLCS are categorized into three dimensions base on a six-point Likert scale scoring system, and the completion time for the scale is approximately 20 min. The total score range of the scale is from 22 to 330, with higher scores indicating better ethical decision-making. There are no cut-off points to classify the respondent's ethical decision-making (22).

### Translation of the scale

Developers of the questionnaire were contacted, and their permission was obtained before translation. The World Health Organization's standard protocol for forward-backward translation was then applied to translate the questionnaire accurately (22, 26). The initial step in translating the NEDM-EOLCS involved two independent translators who translated the English version into Persian in the forward translation phase. Following this, the authors and translators collaborated to agree on a single Persian script for the questionnaire. In the backward translation stage, two additional translators who were not involved in the initial translation process and were unfamiliar with the English version of the questionnaire translated the Persian script back to English. The authors then compared the retranslated English scripts with the original English version, and any discrepancies between the two versions were evaluated throughout the entire process of forward-backward translation. Thirty-two healthcare professionals were randomly selected to offer feedback on the revised Persian version to enhance the scale further. Based on their input, the scale was further revised and improved. Finally, the psychometric properties of the NEDM-EOLCS were assessed.

### Psychometric properties (COSMIN criteria)

#### Face validity

The face validity assessment consisted of two phases qualitative and quantitative. In order in the qualitative phase to ensure the quality of the revised questionnaire, 35 healthcare professionals were tasked with evaluating each item for relevance, appropriate use of grammar and vocabulary, and intelligibility. In the quantitative phase the professionals assessed each item using a 5-point Likert scale ranging from 1 (not important at all) to 5 (very important). Following the evaluation, all questionnaires were collected and analyzed. Any item with an impact score greater than 1.5 was deemed acceptable (24, 26).

#### Content validity

The content validity assessment consisted of two phases qualitative and quantitative. In the qualitative phase the 35 experts to evaluate the NEDM-EOLCS was based on specific inclusion criteria, including having a bachelor's degree, master's degree or PhD degree in nursing and at least one year of professional experience in caring for adults in the end stages of life. The questionnaire was then distributed to 6 nurses with a PhD degree in nursing, 15 nurses with a master's degree in nursing and 14 nurses with a bachelor's degree in nursing from seven different hospitals. These experts assessed each questionnaire item for vocabulary and grammar usage, intelligibility, and relevance to Iranian culture and provided comments for each item. In the quantitative phase after collecting the questionnaires, the experts were asked to assess each item's content validity ratio (CVR), evaluating their usefulness and necessity. The content validity of each item was then measured, and the revised version of NEDM-EOLCS was resubmitted to the 35 participants. They were asked to score each item based on its relevance, simplicity, and clarity, using a four-point Likert scale ranging from 1 to 4. The content validity index (CVI) was then calculated for each item and NEDM-EOLCS. In this study, a CVI score greater than 0.8 and a CVR score greater than 0.31 were considered appropriate (24, 26).

#### Exploratory factor analysis

Exploratory factor analysis was conducted to ensure the NEDM-EOLCS instrument measured what it was intended to measure (27). Varimax rotation was used based on the dimensions of NEDM-EOLCS (28) to achieve an optimal structure. The researchers applied the following criteria: eigenvalues greater than 1.0 and factor loadings greater than 0.05 (29). The adequacy of the samples was evaluated using the Kaiser-Meyer-Olkin (KMO) test for sampling adequacy and Bartlett's test prior to exploratory factor analysis. The KMO value needed to be greater than 0.05 for exploratory factor analysis. If the factor loading for each item was less than 0.5, it was removed from the questionnaire. To assess the construct validity, the ideal sample size was estimated to be 10 times the number of items in the inventory (24, 30, 31).

#### Confirmatory factor analysis

Confirmatory factor analysis was performed on 310 practicing healthcare professionals, who were different from the participants in the exploratory factor analysis. AMOS (v. 21.0) was used for confirmatory factor analysis, and several indices were employed to measure the model's effectiveness. Some requirements, such as a goodness of fit index (GFI) greater than 0.90, a root mean square error of approximation (RMSEA) of less than 0.08, a Tucker Lewis Index (TLI) greater than 0.90, and a comparative fit index (CFI) greater than 0.90 had to be met (24, 31, 32).

#### Reliability (internal consistency and stability)

To measure the reliability of the NEDM-EOLCS, both Cronbach's alpha coefficient and test-retest reliability were used. The Cronbach's alpha coefficient was calculated for 310 samples to evaluate the instrument's internal consistency, with a coefficient greater than 0.7 considered acceptable (31). Test-retest reliability was evaluated using the scale's intra-class correlation (ICC), with data collected from 300 practicing nurses over a two-week interval. An ICC index greater than 0.80 indicated satisfactory consistency of the instrument (24, 30–32).

#### Data analysis

The collected data were analyzed in SPSS 21.0 and AMOS (v. 21.0) using descriptive statistics (frequency, percentage, mean and standard deviation), Cronbach's alpha, test-retest reliability, and factor analysis (24, 31).

#### Ethics approval and consent to participate

The research design was approved by the Ethics Committee of Hamadan University of Medical Sciences (UMSHA.REC.1402.519). At the outset of the study, the researcher introduced herself and explained the study's objectives, ensuring participants that all data would be kept confidential and that they could withdraw from the study at any time. Following this, all participants gave written informed consent after being provided adequate information about the study.

## Results

### Demographic characteristics

The study enrolled participants aged between 28 and 59 years, with a mean age of 41.74 ± 4.38. The majority (68.14%) of the participating heaith care profesional (*n* = 276) had a bachelor's degree in nursing with an average work experience of 11 years by an average monthly income of $700 (Table 1).

**Table 1:** Demographic characteristics (*n* = 310).

Variable		*N* (%)
Gender	Female	165 (53.23)
	Male	145 (46.77)
Marital status	Single	102 (32.90)
	Married	176 (56.77)
	Divorced/Widowed	32 (10.33)
Education level	Bachelor's degree in nursing	142 (45.80)
	Master degree in nursing	127 (40.97)
	PhD degree in nursing	41 (13.23)
Work experience(year)	1–5	65 (20.96)
	6–10	106 (34.20)
	11–15	86 (27.74)
	>15	53 (17.10)
Kind of disease	Cancer	197 (63.55)
	Heart disease	68 (21.93)
	Trauma	45(14.52)

### Face validity

In the qualitative part of face validity, the healthcare professionals stated that the items of this scale have appropriate words and grammar and are simple and understandable. Furthermore, in a quantitative part, all items received an impact score exceeding 1.5, and no item was deleted.

### Content validity

The content validity assessment consisted of two phases qualitative and quantitative. During the qualitative content analysis, 35 healthcare professionals suggested that three of the items (6, 9, 33) in the Persian script needed to be rewritten to enhance the clarity and understanding of their meanings and concepts. After being rewritten, the experts re-evaluated and approved these four items. In quantitative phase the Content Validity Ratio (CVR) was then calculated based on the expert's comments on the necessity of each item, with an acceptable CVR value of 0.31 according to the Lawshe table. All items of the NEDM-EOLCS had a CVR ranging from 0.58 to 1, indicating that none needed to be removed due to unsatisfactory CVR. Additionally, the Content Validity Index (CVI) was calculated for each item and found to range from 0.78 to 1, with none of the items scoring below the cut-off point, and all items were retained. Finally, the Modified Kappa Scale Content Validity Index/Average was determined to be 0.93.

### Exploratory factor analysis

The adequacy of the sample for analysis was demonstrated by the KMO value of 0.92 for the present scale. Furthermore, the factor loadings of the items ranged from 0.68 to 0.89, indicating that no items needed to be removed (Table 2). The factor analysis yielded three factors with eigenvalues greater than 1, which accounted for 82.68% of the total variance (*χ*2 = 3,478.145; *p* < 0.001).

**Table 2:** Varimax factor loadings of the items of the ethical decision-making around end of life care scale for health care professional (*n* = 310).

Factors’ names	Item	Communality	Factor loading
Factor 1: Perceived professional accountability	28- Nurses are responsible for providing the best care for patients at the end of life	0.781	.822
	29- Nurses are responsible for ensuring that a patient's suffering is relieved at the end of life	0.823	.884
	30- Nurses are responsible for assisting patients to make the best healthcare decision	0.801	.852
	31- Nurses are responsible for advocating that a patient's individual needs are met.	0.789	.813
	32- Nurses should ensure patients receive good care even if the patient is difficult or undesirable.	0.672	.739
	33- Nurses are responsible for providing adequate information about the patient's care	0.813	.862
	34- Nurses are responsible for assisting patients to receive hospice or palliative care when invasive interventions are no longer desired or effective.	0.825	.872
	35- Nurses are responsible for their own practice actions.	0.786	.821
	36- Nurses are responsible for recognizing the unethical practice of others and doing something about it	0.757	.816
	37- The nurse should support the patient's reasoned decision to accept or refuse treatment.	0.789	.822
	38- Nurses are responsible for ensuring that patients who have DNR (do-not-resuscitate) order still receive basic nursing care.	0.832	.898
	39- Nurses are responsible for encouraging the patient to be involved in the process of his/her care if the patient is capable.	0.721	.785
	40- Nurses are responsible for advocating that the patient gets what he/she needs even when another nurse, doctor, or family member disagrees with the patients’ considered wishes or desire	0.711	.779
	41- A nurse should refuse to participate in activities that are harmful to the patient	0.787	.812
	42- The nurse should put the patient's safety as the first priority when he/she experiences a conflict with others over the patient's care	0.783	.824
	43- Nurses should use their clinical judgment in deciding whether a treatment or intervention is appropriate for a patient	0.764	.808
	44- All nursing action for a patient should be informed by knowledge, skill, experience, and an understanding of that patient's individual need.	0.753	.803
	45- My actions make a difference to the patient who is facing the end of life.	0.815	.0865
	46- It is meaningful for me to ensure that I care for a patient who is facing the end of life	0.793	.838
	47- It is important that I am sensitive to the individual needs of patients and their family.	0.721	.789
	48- My personal beliefs and values can make me biased toward a particular course of action so I try to understand what these are before acting	0.761	.802
	49- It is important that I remain focused on the responsibility I have toward my patient	0.740	.793
	50- I recognize what the other health professionals’ roles and their responsibilities are.	0.693	.757
	51- Routine nursing and medical procedures have ethical implications for individual patients	0.689	.744
	52- When patients and/or their family are thankful for my actions, it encourages me to persist in getting them what they need.	0.685	.741
	53- When I feel a connection with the patient, I am more likely to act to meet their needs.	0.669	.722
	54- It is my professional responsibility to get my patients needs met even when this is difficult.	0.699	.754
	55- The support of my colleagues helps to keep me focused on getting my patient's needs met.	0.654	.718
Factor 2: Moral reasoning	15- I am able to describe the ethical aspects of a difficult patient situation.	0.825	.879
	16- I can identify when an EOL decision is being made that is not in the interests of the patient.	0.801	.857
	17- I can separate out the barriers to good care in an ethical conflict.	0.793	.838
	18- I know who to go to get help in thinking through a difficult situation	0.786	.824
	19- I feel compelled to act on behalf of my patients when I see they are not getting their needs or wishes met.	0.764	.818
	20- When I am tired or upset, I am still able to focus on meeting my patient's needs in a problematic situation.	0.756	.801
	21- I actively engage in ethical conflict during the EOL care and persist until the patient gets what he/she needs.	0.723	.784
	22- I step back from ethical conflicts and try to think through the issues to find a solution.	0.718	.769
	23- I feel strongly that I must try to resolve an ethical problem even if this is risky for me.	0.710	.759
	24- I confirm the patients’ wishes or preferences regarding DNR/DNI decisions made by family members.	0.698	.763
	25-When institutional policies related to EOL practices are inappropriate, I use current evidence to try to change them.	0.687	.751
	26- I try to ensure that the patient and his/her family are satisfied with their decisions making.	0.657	.723
	27- I confront other healthcare providers when their actions are unethical and might cause harm.	0.635	.684
Factor 3: Moral practice	1- I try to be a comforting presence for the patient who is at the end of life even when he/she does not need hands-on care	0.801	.851
	2- I encourage the patient's family to be with the patient for the final hours.	0.77	.861
	3- I try to tailor care to a patient's individual need.	0.797	.831
	4- I try to help patients at the end of life repair problem relationships they have with important family members or friends.	0.789	.825
	5- I ask the patient what he/she needs related to the dying process.	0.783	.819
	6- I seek out available and current empirical evidence to provide appropriate EOL care to patients.	0.771	.819
	7- I try to help patients find meaning in their condition when they are facing the end of their lives.	0.764	.818
	8- I use knowledge of what actions I would want for my family members to help provide care for the patients.	0.756	.801
	9- I try to mediate between the patient's family and other healthcare providers when there is conflict about the goals of care.	0.740	.791
	10- I try to persuade other healthcare professionals and the patients’ family to honor the patient's wishes when they are acting contrary to what the patient wants.	0.736	.788
	11- I try to provide education to the patient and family about the purpose of any technology or therapies being used.	0.723	.782
	12- I provide appropriate information about the purposes and goals of withdrawing or withholding treatment	0.710	.757
	13- I try to understand what the patient's preference regarding EOL care is and to advocate for this to be heard by those making the decisions.	0.667	.742
	14- I try to meet with the patient's family regularly and answer their questions.	0.631	.681

### Confirmatory factor analysis

**Figure 1:**
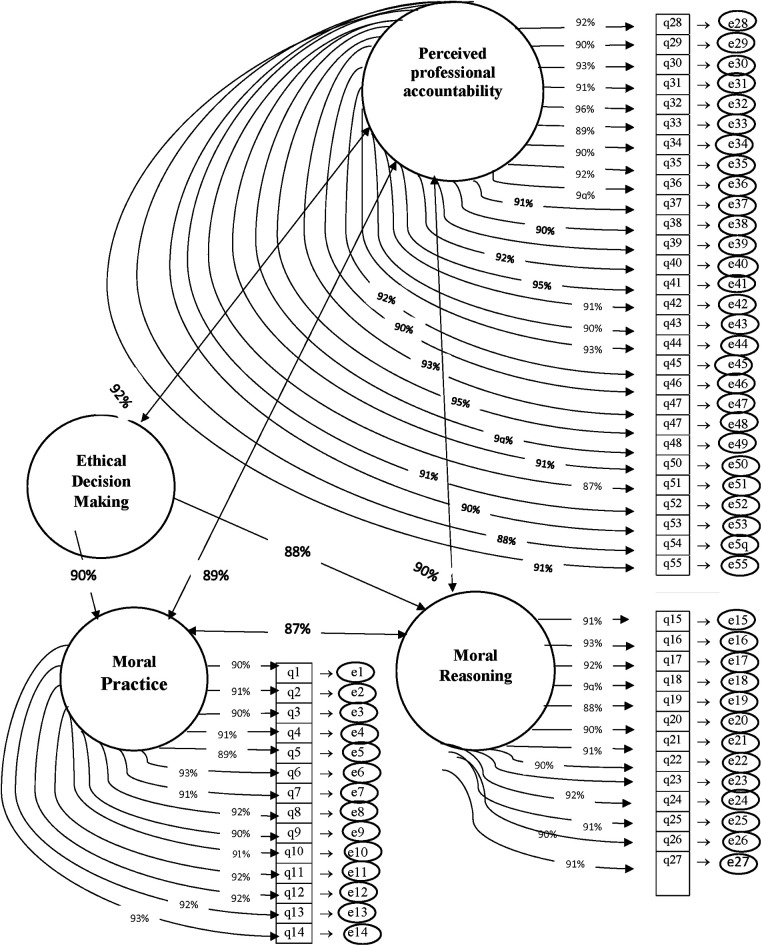
Model fit the Decision-Making in End-of-Life Care Scale.

The confirmatory factor analysis results indicated a model with three factors, namely professional accountability (28 items), moral reasoning (13 items), and moral practice (14 items). The correlation of factors 1, 2, and 3 with the entire instrument was 0.92, 0.91, and 0.90, respectively. Moreover, a chi-square value of 15.74 (df = 8, *p* = 0.001) demonstrated good fitness of the model. The Goodness of Fit Index (GFI) value was 0.92, indicating that the uni-dimensional model of the PTES constructs fit well in the present study. Other indices tested in this model were RMSEA = 0.01, CFI = 0.92, NFI = 0.93, and TLI = 0.92. All tested indices demonstrated that the extracted model fit well (Figure 1).

### Reliability

The questionnaire's reliability was evaluated using Cronbach's alpha coefficient and test-retest reliability. The internal consistency of the 55-item instrument was found to be 0.932 using Cronbach's alpha coefficient. Moreover, the three subscales of “professional accountability,” “moral reasoning,” and “moral practice” had internal consistency coefficients of 0.953, 0.925, and 0.91, respectively (Table 3).

**Table 3:** Cronbach's alpha of subscales and the entire the ethical decision-making around end of life care scale.

Factors	Subscale	Items	Cronbach's alpha
1	Perceived professional accountability	28	0.953
2	Moral reasoning	13	0.925
3	Moral practice	14	0.918
Total		36	0.932

To assess the test-retest reliability of the questionnaire, 80 healthcare professionals were invited to complete the questionnaire after a two-week interval. The test-retest analysis revealed no statistically significant difference between pre, and post-test scores (*p* = 0.67). The correlation coefficients between the scores on the professional accountability subscale in the test-retest were 0.94, while those for the moral reasoning and moral practice subscales were 0.95 and 0.92, respectively. Finally, the correlation coefficient of the test-retest was 0.93, indicating the instrument's stability (Table 4).

**Table 4:** Mean (standard deviation) and intraclass correlation coefficient (ICC) values for the domains of the ethical decision-making around end of life care scale.

Factor	Dimensions	Mean ± SD	ICC	Confidence interval	*p*-value
1	Perceived professional accountability	124.87 (3.56)	0.94	0.78- 0.98	*p* < 0.05
2	Moral reasoning	54.82 (3.25)	0.95	0.87–0.97	*p* < 0.05
3	Moral practice	48.67 (3.12)	0.92	0.83–0.94	*p* < 0.05
Total		195.92 (3.31)	0.93	0.82–0.93	*p* < 0.05

## Discussion

The present study was conducted to translate and evaluate the Persian version of the Moral Decision-Making in End-of-Life Care Scale for healthcare professionals in Iran. Notably, the Moral Decision-Making in End-of-Life Care Scale was originally designed and validated only in Korea. Given the absence of relevant studies, the researchers compared their findings with those of a previous study conducted by Kim in 2010.

The nurses in this study were Muslims. In Iranian culture, ethical care is performed based on Islamic principles. However, individual, ethnic, religious and cultural differences can strongly affect the moral care of patients in the final stages of life (24). One of the important ethical issues in end-of-life care is euthanasia. In Iran, non-voluntary active euthanasia is unethical and illegal, and the request for voluntary passive euthanasia is only considered in cases of incurable diseases and very critical patients with the opinion of a few specialized and committed doctors (33). Based on this, the questions related to participation in end-of-life care decision-making in qualitative content validity were reviewed and simplified with experts' opinions.

The research findings indicated that as same as the original version of the scale, the Persian version of the Moral Decision-Making in End-of-Life Care Scale for healthcare professionals demonstrated satisfactory validity and reliability, and none of the 55 questionnaire items were removed. The face validity assessment demonstrated that all 55 items had a factor loading of over 1.5, and none were removed. Moreover, the content validity assessment revealed that the CVR of each item ranged from 0.58 to 1, indicating a satisfactory level of agreement. The I-CVI of the scale was between 0.78 and 1, and the S-CVI was 0.93, indicating a good level of satisfaction. Consistent with the present study, the content validity assessment in Kim's study (2010) also demonstrated an acceptable and suitable content validity for the Moral Decision-Making in End-of-Life Care Scale (22). However, the CVR and S-CVI were not reported in that study.

In the present study, exploratory factor analysis demonstrated a KMO of 0.92, and the three factors explained 81.64% of the variance, with factor loadings ranging from 0.68 to 0.89, indicating satisfactory satisfaction. Similarly, Kim's study (2010) showed a KMO of 0.91. After conducting exploratory factor analysis, the three subscales of the Korean version of the Moral Decision-Making in End-of-Life Care Scale accounted for 44.50% of the variance, with factor loadings ranging from 0.45 to 0.76, which were considered satisfactory (22), and are consistent with those of the present study.

The confirmatory factor analysis in the present study showed acceptable model fit indices, with an average variance ranging from 0.63 to 0.93. Kim's study did not report the confirmatory factor analysis results (22). Moreover, it was found that the Persian version of the Moral Decision-Making in End-of-Life Care Scale is reliable and satisfactory, with Cronbach's alpha coefficients ranging from 0.91 to 0.95 for the three subscales and an overall Cronbach's alpha coefficient of 0.93. The total scale's intraclass correlation coefficient (ICC) was also satisfactory at 0.93. Similarly, Kim's study (2010) reported satisfactory reliability for the Korean version of the scale, with Cronbach's alpha coefficients of 0.95, 0.88, and 0.89 for the three subscales and an ICC of 0.95 for the total scale. These findings are consistent with those of the present study (22).

## Limitations

The target population of the present study consisted of healthcare professionals in government hospitals. Therefore, it is recommended that future research also include healthcare professionals in private hospitals. The present study did not address the factors influencing ethical decision-making in end-of-life care, as it was not one of the study objectives. It is suggested that future research investigate these factors as well. Additionally, given the cultural differences among different countries, this scale should be translated and evaluated in other countries. As the Moral Decision-Making in End-of-Life Care Scale had only been translated and evaluated in Korea, the researchers in the present study could only compare their findings with those of Kim's study, which is another limitation of the present study.

## Conclusion

The Iranian version of the Moral Decision-Making in End-of-Life Care Scale is sufficiently reliable and valid. As a result, those in charge of health policy and management can employ this tool to assess the ethical decision-making abilities of healthcare professionals in end-of-life care settings. Additionally, it is recommended that nursing educators integrate teaching and evaluation of ethical decision-making in end-of-life care into their curriculum for nursing students. All in all, the findings of this study could prove to be a valuable resource for developing and evaluating the effectiveness of an ethical decision-making program targeted toward improving end-of-life care.

## Data Availability

The original contributions presented in the study are included in the article/Supplementary Material, further inquiries can be directed to the corresponding author.
